# Oral administration of *Sophora Flavescens*-derived exosomes-like nanovesicles carrying CX5461 ameliorates DSS-induced colitis in mice

**DOI:** 10.1186/s12951-024-02856-z

**Published:** 2024-10-08

**Authors:** Manqi Zhang, Xichao Xu, Liqian Su, Yuqing Zeng, Jingxiong Lin, Wenwen Li, Yigui Zou, Sicong Li, Boxian Lin, Ziyuan Li, Hu Chen, Yuheng Huang, Quanle Xu, Hongbo Chen, Fang Cheng, Dongling Dai

**Affiliations:** 1 Endoscopy Center and Gastroenterology Department, Key Laboratory for Precision Diagnosis and Treatment of Pediatric Digestive System Diseases, Shenzhen Children’s Hospital, Shenzhen, 518036 China; 2 School of Pharmaceutical Sciences (Shenzhen), Shenzhen Campus of Sun Yat-sen University, Gongchang Road, Shenzhen, Guangdong 518107 China; 3 Brain Cognition and Brain Disease Institute, Shenzhen Institute of Advanced Technology, Chinese Academy of Sciences, Shenzhen, 518052 China; 4 College of Life Sciences, Northwest A&F University, Yangling, Shaanxi 712100 China

**Keywords:** Ulcerative colitis, *Sophora Flavescens*-derived exosomes-like nanovesicles, CX5461, Inflammation, Macrophage

## Abstract

Ulcerative colitis (UC) belongs to chronic inflammatory disease with a relapsing characterization. Conventional oral drugs of UC are restricted in clinical by premature degradation in the gastrointestinal tract, modest efficacy, and adverse effects. CX5461 can treat autoimmune disease, immunological rejection, and vascular inflammation. However, low solubility, intravenous administration, and non-inflammatory targeting limited its clinical application. Herein, this work aims to develop *Sophora Flavescens*-derived exosomes-like nanovesicles carrying CX5461 (SFELNVs@CX5461) for efficient CX5461 oral delivery for UC therapy. We identified SFELNVs as nano-diameter (80 nm) with negative zeta potential (-32mV). Cellular uptake has shown that SFELNVs were targeted uptake by macrophages, thus increasing drug concentration. Additionally, oral SFELNVs@CX5461 exhibited good safety and stability, as well as inflammation-targeting ability in the gastrointestinal tract of dextran sodium sulfate (DSS)-induced colitis mice. In vivo, oral administration of SFELNVs and CX5461 could relieve mice colitis. More importantly, combined SFELNVs and CX5461 alleviated mice colitis by inhibiting pro-inflammatory factors (TNF-α, IL-1β, and IL-6) expression and promoting M2 macrophage polarization. Furthermore, SFELNVs promoted M2 polarization by miR4371c using miRNA sequencing. Our results suggest that SFELNVs@CX5461 represents a novel orally therapeutic drug that can ameliorate colitis, and a promising targeting strategy for safe UC therapy.

**Supplementary Information:**

The online version contains supplementary material available at 10.1186/s12951-024-02856-z.

## Introduction

Ulcerative colitis (UC) belongs to inflammatory bowel disease (IBD), is a chronic and multiple factors-induced inflammatory intestinal disease affecting human health worldwide [1]. UC has the characteristics of bloody diarrhoea, dysregulated immune response, pain, and weight loss in clinical symptoms [2], together with increased-cancer risk [1]. Studies have found that patients with extended UC could increase the risk of colorectal cancer (CRC). In China, the incidence of patients with UC-related CRC has been increasing [3]. Currently, the therapeutic drugs include immune inhibitors, steroids, sulfasalazine (SASP), and other anti-inflammatory reagent [4, 5], are not efficient thoroughly and exist some side effects, such as hepatotoxicity and headache [6–9]. Therefore, a novel therapeutic strategy for UC treatment is imminently needed.

Macrophages play important roles in inflammation and intestinal homeostasis [10]. In normal conditions, macrophages are distributed and enriched in the whole intestinal tissue to inhibit harmful bacteria-triggered inflammation [11]. M2 subtypes of macrophages produced chemokines and cytokines to maintain intestinal homeostasis and repair mucosal barrier when the bowel barrier was impaired [12]. In the IBD mice model, the numbers of pro-inflammatory macrophages (M1) were significantly elevated [13, 14], as well as in IBD patients [14, 15]. Furthermore, these macrophages also increased the pro-inflammatory factors expressions, such as IL-6, iNOS, IL-1β, and TNF-α [16, 17]. In inflamed intestine, M1 pro-inflammatory macrophages disrupted the gut epithelial barrier and the tight junction proteins, contributing to excessive gut inflammation. More importantly, the expressions of lasting M1 pro-inflammatory phenotypes have been demonstrated to exacerbate the inflammation and induce the gut damage [18]. Thus, targeted inflammatory site and inhibition of inflammation is a potential strategy for UC treatment. Based on the concept, anti-TNF-α is a potent drug for UC treatment. However, administered systemically and serious adverse effects limits its application [19].

CX5461 is the RNA polymerase (Pol I) inhibitor that can against cancers, and have an excellent safety in clinical research [20, 21]. Recently, CX5461 was reported to suppress macrophage-mediated inflammation of vascular [22], and inhibit imiquimod-induced psoriasis and inflammation in mice models and decrease the T infiltration [23]. In the previous research of our groups, they were the first to discover CX5461 as an immunosuppressant by repressing the transcription of NF45/NF90-dependent rDNA in mice skin and heart transplantation models [24]. Mechanistically, CX5461 alleviated immune rejection through blocking NFAT-mediated T cells activation pathway. Compared to clinical drug, FK506, CX5461 could effectively prolong the survival time of the transplant and had lower side effects [24]. In addition, they also found that CX5461 could inhibit CD4^+^T cells proliferation in vitro and infiltration in vivo, thus relieving DNCB-induced atopic dermatitis [25]. These results implying that CX5461 can treat inflammatory diseases or immune diseases. UC are driven by immune cells dysregulation and the production of excess inflammation factors. Hence, we speculated that CX5461 might have a treatment effect on UC owing to its function of anti-inflammation and immunoregulation. Interestingly, in our preliminary study, we found that CX5461 could inhibit M1 macrophages proliferation and promote M1 macrophages apoptosis, indicating that CX5461 indeed regulated inflammation.

For the UC clinical therapy, oral administration is the optimal strategy for UC patients due to its convenience, patient compliance, and direct drug delivery to the inflamed intestine tissue [26, 27]. Unfortunately, low solubility, instability under physiological conditions, and intravenous administration in clinical trial of CX5461, even the injected compound of CX5461 may produce precipitates in physiological pH [28], resulting in reduced treatment efficacy and limited clinical application. Therefore, improving the stability of CX5461 and inflammatory targeting capacity to enhance therapeutic efficacy of UC is imperative. Recently, artificial nanoparticles with targeting inflammatory ability were widely used for UC treatment [29–31]. However, these nanoparticles are limited by potential tissue toxicities and expensive in clinical [32–34]. Excitedly, plant-derived exosomes-like nanoparticles (PELNPs) serve as novel drug delivery systems for UC treatment. They are characterized by oral administration, excellent biocompatibility and safety, easy availability, and stability [32, 35, 36]. Thus, PELNPs could protect CX5461 from degrading in the gastric acidic microenvironment. More importantly, PELNPs could localize to the inflamed colon site, such as turmeric-derived exosome-like nanovesicles, preferentially located in the inflamed gut after oral administration, although the underlying mechanism was not clear [32], thus increasing CX5461 concentration in inflamed colon and enhancing therapy efficacy. Additionally, PELNPs have many functions, including immunological modulation, anti-tumor activities, regenerative effects, and anti-inflammatory actions [32, 35]. For example, orally administrated turmeric-derived nanovesicles could decrease mice colitis through inhibiting pro-inflammatory factors (IL-6, IL-1β, and TNF-α) expression [32]. Edible-derived nanovesicles ameliorate mice colitis via decreasing pro-inflammatory cytokines expression and promoting CD4^+^CD8^+^T cells activation [36]. These advantages and functions make PELNPs become a promising oral drug delivery system to target the inflamed colon tissue to enhance therapeutic effects. In our preliminary study and literature research, we screened five traditional Chinese medicine, including *Sophora Flavescens*, *Codonopsis Pilosula*, *Astragalus Membranaceus*, *Andrographis Paniculate*, and *Bupleurum Chinense*. Their anti-inflammatory function has been demonstrated [37–41]. We speculate that these five traditional Chinese medicine-derived exosomes-like nanovesicles have the similar function of theirs. Five traditional Chinese medicine derived exosomes-like nanovesicles were extracted and purified by ultracentrifugation. We investigated their stability in gastrointestinal simulation and cell uptake by Caco-2 enterocyte. *Sophora Flavescens*-derived exosomes-like nanovesicles (SFELNVs) showed excellent stability and uptake by Caco-2 cells. Besides, the loading efficacy of CX5461 into SFELNVs was 23%, thus improving CX5461 solubility and stability.

**Scheme 1:**
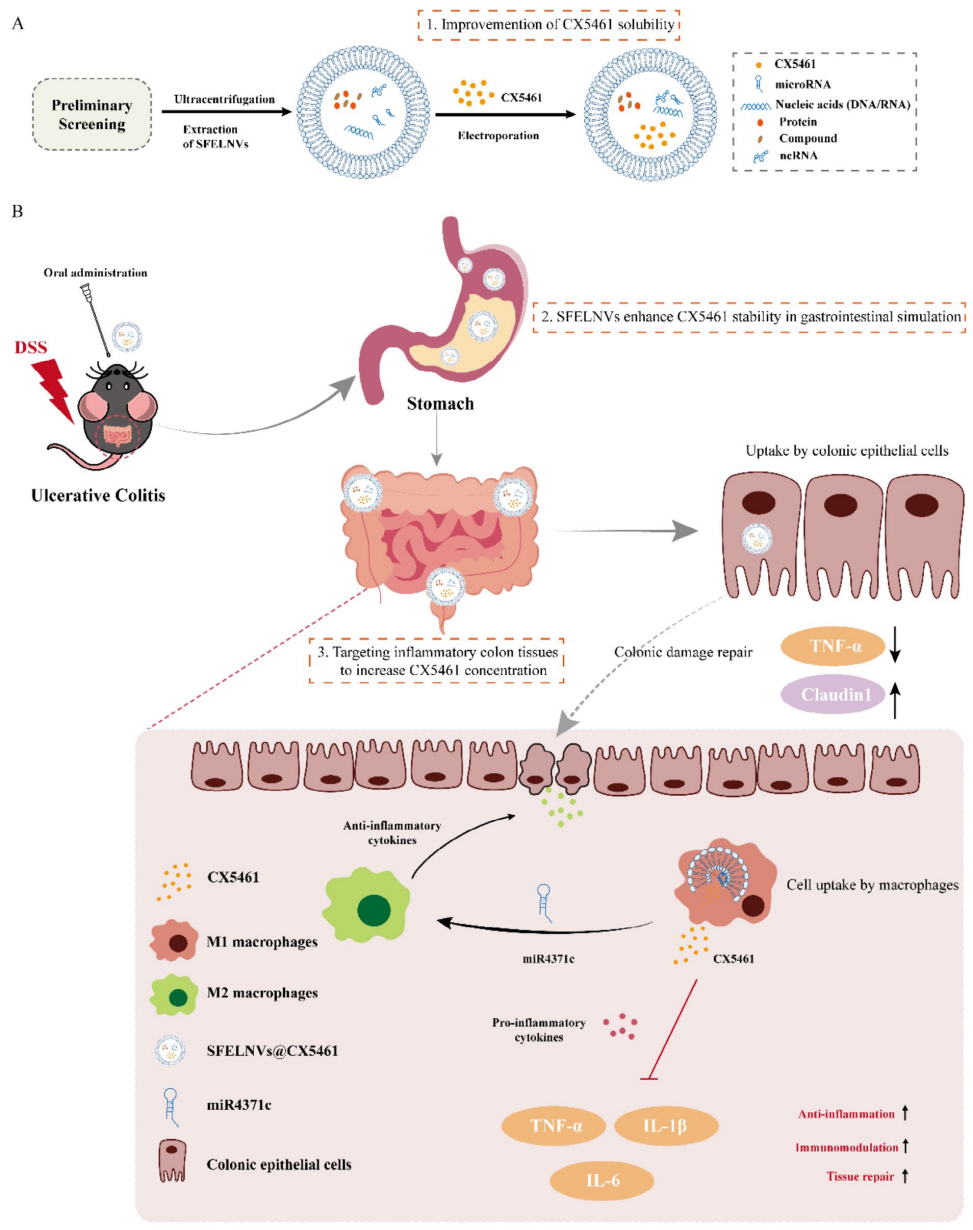
Schematic illustration of oral administration of SFELNVs carrying CX5461 for the treatment of DSS-induced mice colitis. (**A**) Preparation of SFELNVs@CX5461. (**B**) Orally administrated SFELNVs@CX5461 treat DSS-induced mice colitis. (1) Traditional Chinese medicines-derived exosomes were screened in the gastrointestinal simulator and cell uptake by Caco-2. (2) Orally administrated SFELNVs@CX5461 was taken up by macrophages and intestinal epithelial cells, and released CX5461 in the flamed colon tissues. (3) CX5461 inhibited pro-inflammatory factors expression, and miR4371c repressed M1 polarization, thereby promoting UC treatment

To address the clinical need, we propose a novel stable and inflammation-targeted platform (SFELNVs@CX5461) to effectively oral delivery anti-inflammation drugs. Scheme 1 exhibits the hypothesis in our study. The delivery strategy of PELNPs@CX5461 are characterized by oral administration, targeting inflammatory colon tissues to increase CX5461 concentration, and improve CX5461 stability. First, SFELNVs showed excellent stability in gastrointestinal simulation. SFELNVs and CX5461 could inhibit M1 macrophages proliferation. In addition, SFELNVs facilitated M2 macrophages polarization through miR4371c, as well as downregulated pro-inflammatory expression. Then, CX5461 was encapsulated into SFELNVs using electroporation, thus enhancing CX5461 stability, solubility, and inflammatory targeting. In DSS-induced colitis mice, oral administration of SFELNVs mainly located in inflamed colon and were taken up by macrophages. Orally administrated SFELNVs@CX5461 could attenuate DSS-induced colitis and repress pro-inflammatory factors expression, thereby alleviating the inflammation of UC mice. Therefore, SFELNVs@CX5461 serve as a novel therapeutic strategy for UC with inflammatory targeting and excellent stability.

## Materials and methods

### Cell culture

RAW264.7 and Caco-2 cells were cultured in Dulbecco’s Modified Eagle’s Medium (Gibco, CA, USA) supplemented with 10% fetal bovine serum (FBS) (ExCell Bio, Canelones, Uruguay).

### Chemicals and regents

CX5461 (Biochempartner, #1138549-36-6), sulfasalazine (SASP) (Sigma, #599-79-1) was used for UC treatment. LPS (Sigma, #297-473-0) was used for the induction of macrophage inflammation. DSS (MP Biomedicals, Cat#160110) was constructed mice colitis. Mouse TNF-α ELISA kit (Dekewe, #2208-1), mouse IL-6 ELISA kit (Dekewe, #2207-2), and mouse IL-1β ELISA kit (Dekewe, #2304-1) were used for ELISA according to the instructions. Anti-CD11b (BioLegend, Cat#101205), F4/80 antibody (BioLegend, Cat#123115), anti-CD80 (BioLegend, Cat#104725), anti-CD86 (BioLegend, Cat#159203), anti-CD86 (BioLegend, Cat#105012), anti-CD206 (BioLegend, Cat#141717), anti-CD206 (BioLegend, Cat#141707) were prepared for flow cytometry. DiO (Bioss, D-9102) and DiR (Bioss, D-9111) for labeling the nanovesicles. Annexin V/PI (Elabscience, AK11013) were prepared for apoptosis detection by flow cytometry. TNF-α (Servicebio, GB11188) and Occludin (ProteinTech, 27260-1-AP) was used for IHC staining. The primers of qPCR were list in the Table 1.

**Table 1:** The sequences of q-PCR primer and miR-4371c

Gene name (Mouse)	Forward primer (5’-3’)	Reverse primer (5’-3’)
*iNOS*	GTTCTCAGCCCAACAATACAAGA	GTGGACGGGTCGATGTCAC
*IL-1β*	GAAATGCCACCTTTTGACAGTG	TGGATGCTCTCATCAGGACAG
*IL-6*	TCTATACCACTTCACAAGTCGGA	GAATTGCCATTGCACAACTCTTT
*TNF-α*	CTGAACTTCGGGGTGATCGG	GGCTTGTCACTCGAATTTTGAGA
*β-actin*	GTGACGTTGACATCCGTAAAGA	GCCGGACTCATCGTACTCC
miR-4371c	GACGUGACAGACGGAAUAUCACAU	

### SFELNVs extraction

Plant *Sophora Flavescens* was homogenized using a juice extractor followed by the ultracentrifugation. Then, centrifugation at 500 g, 2000 g, 10,000 g, and 40,000 g at 4 °C for 60 min, and 150,000 g was performed for 120 min. After that, SFELNVs were obtained and stored at − 80 °C.

### Characterization of SFELNVs

SFELNVs were dissolved in PBS, and then filtered through 0.1 μm filters. The morphology of SFELNVs were charactered by transmission electron microscopy (TEM). The size diameter and zeta potential of SFELNVs were measured via the dynamic light scattering (DLS) and nanoparticle tracking analysis (NTA). The concentration of SFELNVs were evaluated through NTA.

### Cellular uptake assay

5 µM DiO staining solution was mixed with SFELNVs and incubated at 37℃ for 15 min. Subsequently, the remaining dye was removed with ultrafiltration for 10 min. After that, co-incubation of RAW264.7 cell lines and 50 µg DiO-labeled SFELNVs was performed for 0, 6,12, 24, 48, and 72 h. Caco-2 cells were treated by 100 µg/mL DiO-labeled SFELNVs for 0, 6,12, 24, and 48 h at 37℃. After harvesting the cells and washing by PBS, the fluorescence intensity was investigated using flow cytometry. In addition, DiO-labeled SFELNVs and RAW264.7 cells were co-incubated in medium for 24 h. After fixation and washing, the fluorescence images were obtained through confocal microscopy.

### Preparation of SFELNVs@CX5461

The SFELNVs and CX5461 were homogeneous mixed in the electroporation solution, and then they were electroporated using the electroporator (Bio-Rad) at 4℃ conditions of 300 V and 150µF for 30 min treatment. Then, washing by PBS and centrifugation for 1 h, SFELNVs encapsulated with CX5461 were obtained. The loading efficiency of SFELNVs@CX5461 were calculated by UV-spectrophotometer.

### Proliferation and apoptosis experiments

Cell proliferation was detected by the CFSE kit (BioLegend, #423801). According to the instruction of the kits, mixing the 10^8^ cells/mL in the CFSE working solution (5µM), and then the cells were incubated for 20 min at 37℃ away from light. Then, adding 5 times staining volume of complete medium and incubating the cells for 10 min continuously. After incubation, cells were treated by SFELNVs or CX5461 and assessed the cell proliferation by flow cytometry.

Cell apoptosis was detected by the Annexin V/PI apoptosis detection kit (Abbkine, #KTA0002). In brief, RAW264.7 was treated by SFELNVs or CX5461, and then harvesting the cells and staining by the Annexin V/PI apoptosis detection kit, and detecting the cell apoptosis by flow cytometry.

### ELISA experiments

The protein expressions of inflammatory cytokines were assessed by ELISA kits. According to the instruction of the kits, 100µL sample was added in sample well. Adding 50µL 1×biotinylated antibody and incubated them at 25℃ for 60 min. After incubation, adding 300µL/well 1×Washing Buffer to wash the well, repeat 3 times. Then, 100µL 1×Streptavidin-HRP was added and incubated at room temperature for 20 min. Washing the well by 1×Washing Buffer. After that, 100µL TMB was performed for developing for 10 min. Quench the reaction by adding 100µL Stop Solution, and then measured under 450 nm wavelength.

### LC-MS and MicroRNA sequencing assay of SFELNVs

50µL of the SFELNVs were diluted with 1mL methanol solution, and then filtered into sample vial using 0.1 μm filter. The sample was put into the LC-MS sample tray with 2µL the sample volume and the LC flow rate was 0.5mL/min (Spray voltage: 4000 V; auxiliary gas: 10 arbitrary unit; sheath gas: 40 arbitrary unit). Mobile phase: pH = 10, H_2_O: MeOH (95:5). The temperature of column is 24℃. Then, the components in the SFELNVs were assessed using LC-MS assay.

Plant *Sophora Flavescens* was homogenized using a juice extractor, and SFELNVs were extracted using the ultracentrifugation. Then, three SFELNVs samples were performed miRNA sequencing by Lifeint company (Xiamen, China). After miRNA sequencing, miR4371c was the most enrich miRNA in SFELNVs. miR4371c mimic was synthesized from Genepharma. The sequences of miR-4371c mimic were list in the Table 1.

When RAW264.7 cells reached 70-80%, transfection of NC (50nM) and miR4371c mimic (50nM) were performed by GP-transfect-mate agents (Genepharma) in LPS-induced cells. After 24 h treatment, flow cytometry was used to investigate the expression of CD86 and CD206.

### Animal experiments

To establish the colitis model of mice, C57BL/6 mice (8 weeks, 18–20 g, BesTest Bio-Tech Co.,Ltd, Zhuhai) were induced by 3% DSS in drinking water for a week. To assessed the treatment efficacy of SFELNVs@CX5461, DSS-induced C57BL/6 colitis mice models were orally administrated with SFELNVs@CX5461 (*n* = 5, 80 mg/kg), SFELNVs (*n* = 5, 80 mg/kg), and SASP (*n* = 5, 300 mg/kg) for 5 days. The body weight, consistency of stool, and rectal bleeding were measured each day. Lastly, after sacrificing the mice, colons and main organs were obtained for the qPCR, HE, and IHC.

### In vivo imaging system assay of SFELNVs distribution

5 µM DiR staining solution was mixed with SFELNVs and incubated at 37℃ for 15 min. Subsequently, the remaining dye was removed with ultrafiltration for 10 min. After that, either DiR-labeled SFELNVs solution or diluted DiR fluorescent dye (as control) was orally administered into 3% DSS-induced UC model mice on day 6. The groups are NC + DiR-SFELNVs, UC + DiR, and UC + DiR-SFELNVs. The distribution of SFELNVs in the mice was captured using a living imaging instrument at different time points (6, 12, 24, and 48 h). thereafter, the main organs (heart, liver, spleen, lung, and kidney) and the intestine were harvested. Subsequently, the distribution and retention of SFELNVs in the gastrointestinal tract and the main organs were investigated using a living imaging system.

### Hematoxylin & eosin staining

Hematoxylin & Eosin staining (H&E) were performed according to the instructions of the kits (Beyotime, C0105M, China). In brief, after deparaffinization by xylene and hydration using distilled water, main organs tissues and colons from mice were stained using H&E reagent. Finally, the images of main organs and colon tissue sections were obtained by microscopy.

### Immunohistochemistry staining

Immunohistochemistry staining (IHC) was displayed using a kit (Zhongshan golden bridge biotechnology, #PV-9001, China). After deparaffinization and hydration, colon tissues from mice were incubated with anti-TNF-α and anti-occludin overnight at 4℃. Then, the sections were incubated with secondary antibody followed by a DAB kit. The images of immunohistochemistry staining sections were obtained by microscopy.

### Statistical analysis

All statistical data were performed by GraphPad Prism9.0.0. All *p*-values were calculated via t-test analysis. Data are performed as mean ± SD. *, *p* < 0.05; **, *p* < 0.01; ***, *p* < 0.001, and ns, not significant. All the showed data were repeated at least three times.

## Results

### Characterization of *Sophora Flavescens*-derived exosomes-like nanovesicles (SFELNVs)

To screen the plant-derived exosomes-like nanoparticles (PELNPs) with good therapeutic effect and stability on UC, we selected five traditional Chinese medicines known to be effective in treating inflammation, including *Sophora Flavescens*, *Codonopsis Pilosula*, *Astragalus Membranaceus*, *Andrographis Paniculate*, and *Bupleurum Chinense*. PELNPs were homogenized using a juice extractor followed by the ultracentrifugation method as described in materials and methods section. To explore the oral stability of five PELNPs, we simulated the PELNPs in simulation of gastric fluid (SGF), simulation of intestinal fluid (SIF), and simulation of colonic fluid (SCF) in vitro, followed by monitoring their average diameter. We found that *Sophora Flavescens*-derived exosomes-like nanovesicles (SFELNVs) had better stability (Extended data Fig. 1). Moreover, we investigated the cell uptake of five PELNPs in Caco-2 cells. Flow cytometry analysis showed that the five PELNPs-labeled with the DiO was taken up by Caco-2 cells, the quantified fluorescence intensity of SFELNVs, CPELNVs, AMELNVs, APELNVs, and BCELNVs was 79.74%, 69.56%, 40.44%, 40.64%, and 39.36% at 12 h, respectively (Extended data Fig. 2). There results indicated that the SFELNVs could be taken up effectively by Caco-2 cells. Thus, we determined the SFELNVs to serve as delivery system for UC treatment. We further monitored the components in the SFELNVs via LC-MS. LC-MS results showed that SFELNVs contained 7,11-Dehydrooxymatrine, Kushenol, Anagyrine, Liquiritigenin, and Sophoraflavanones, which were involved in anti-inflammatory response (Extended data Fig. 3A). Furthermore, we analyzed the common target genes and pathways of the components of SFELNVs and UC through network pharmacology. Using TCMSP online database, we identified 111 common genes in SFELNVs and UC-related genes (Extended data Fig. 3B). PPI protein interaction analysis revealed that numerous significant genes were associated with UC, and the top fifteen genes, TNF, AKT1, MAPK, MTOR and STAT1, were crucial for the progression of UC (Extended data Fig. 3C and D). GO and KEGG enrichment showed that the common genes mainly enriched in inflammatory pathway including inflammatory response, inflammatory mediator regulation of TRP channels, MAPK, Th17, Toll-like receptor signaling pathway, JAK-STAT signaling pathway, and PI3K-akt pathway (Extended data Fig. 3E and F), showing that SFELNVs could involve inflammatory response. Therefore, these results indicating that SFELNVs could play the role in inhibiting inflammation.

**Fig. 1:**
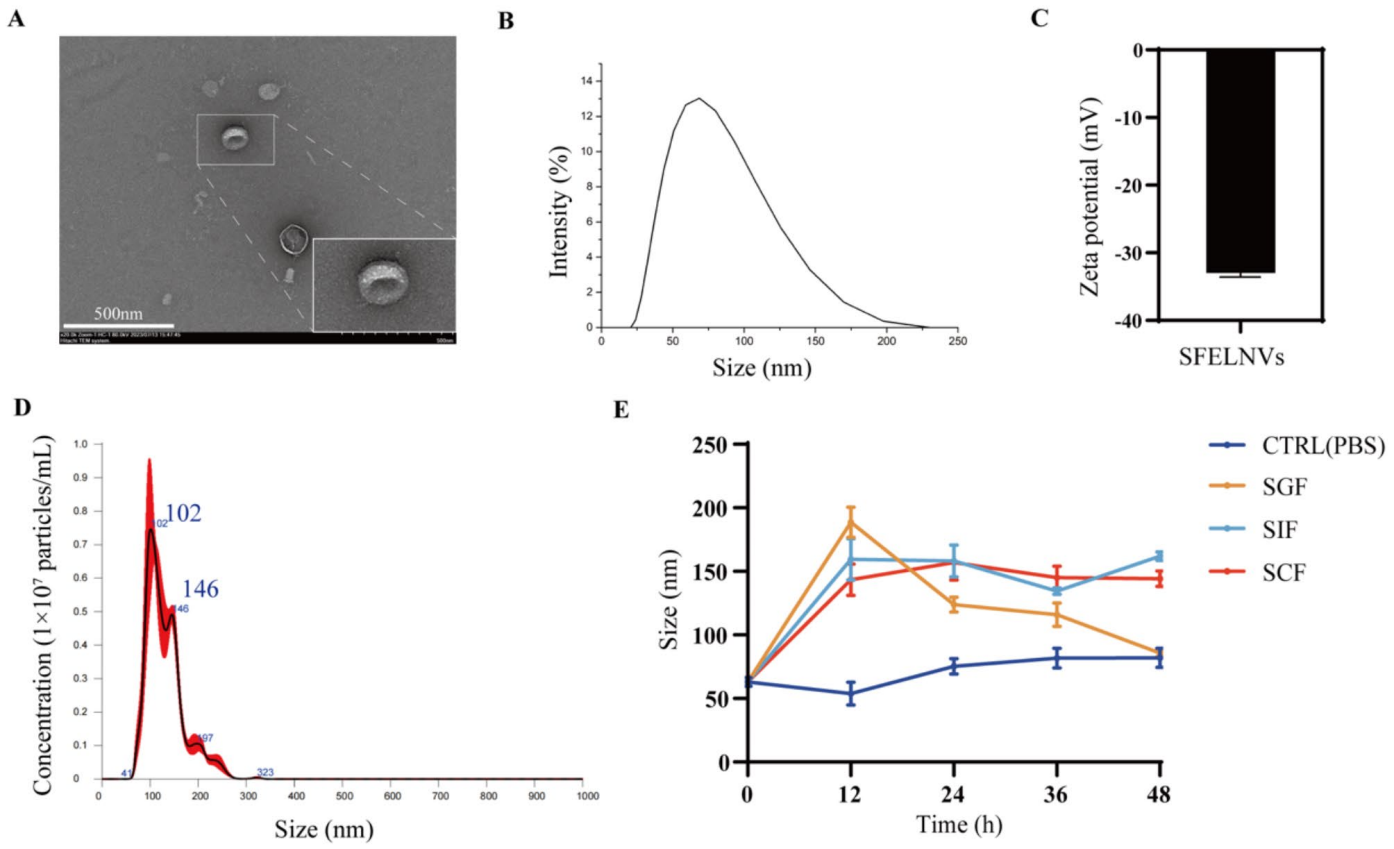
Characterization of SFELNVs. (**A**)TEM images to characterize the size and morphology of SFELNVs. Scale bar, 500 nm. (**B**) The particle size distribution of SFELNVs was determined by the DLS. (**C**) Zeta potential of SFELNVs (*n* = 3). (**D**) Representative particle concentration and size distribution of SFELNVs by NTA. (**E**) Stability analysis of SFELNVs in SGF, SIF, and SCF (*n* = 3)

To investigated the functions of the SFELNVs, we firstly extracted and characterized the SFELNVs. Multiple SFELNVs have been characterized by TEM and DLS examination. As shown in Fig. 1A, the morphology of SFELNVs possessed a hemispherical or ellipsoid-shape with a concave side using the TEM examination. As detected using DLS method, the average diameter of SFELNVs was about 80 nm (Fig. 1B). The zeta potential of SFELNVs was around − 32 mV (Fig. 1C). Meanwhile, as measured by nanoparticle tracking analysis (NTA), the nanoparticle concentration of SFELNVs was about 8 × 10^6^/mL. We also found that the average diameter of SFELNVs was about 100 nm (Fig. 1D), which was similar to the results of DLS method. Furthermore, in order to evaluate the stability of SFELNVs, we treated the SFELNVs in SGF, SIF, and SCF, followed by monitoring their average diameter. Results have shown that the particles size of SFELNVs was about 80 nm at 24 h after treated in SGF, while the particles size of SFELNVs was 150 nm in SIF and SCF treatment (Fig. 1E). The change of particles size did not affect the functions, indicating that SFELNVs possessed a favorable stability. Therefore, SFELNVs could serve as an excellent oral delivery platform.

### SFELNVs could be taken up by macrophages and mainly located in the inflamed colon

**Fig. 2:**
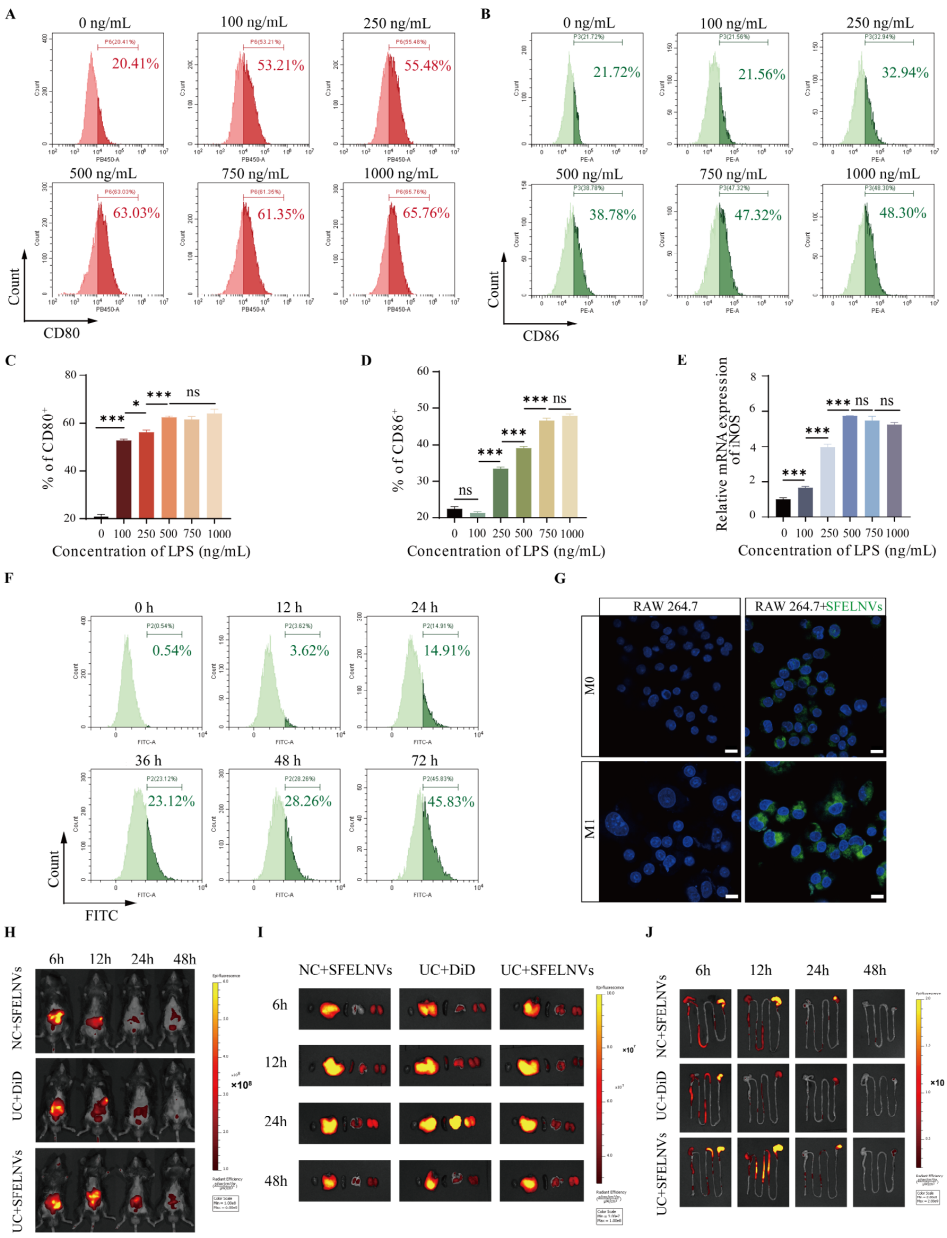
Investigation of the targeting ability of SFELNVs in vitro and in vivo. (**A**) CD80 expression of RAW264.7 was detected using flow cytometry after LPS induction at different concentrations. (**B**) Quantitative analysis of CD80 expression. (**C**) CD86 expression of RAW264.7 was detected using flow cytometry after LPS induction at different concentrations. (**D**) Quantitative analysis of CD86 expression. (**E**) iNOS mRNA expression in RAW264.7 was detected by qPCR after LPS induction at different concentrations. *n* = 3, *, *p* < 0.05; ***, *p* < 0.001, and ns, not significant. (**F**) The cellular uptake efficiency of SFELNVs by RAW264.7 was detected using flow cytometry. (**G**) Representative images of SFELNVs cellular uptake by M0 and M1 phenotype RAW264.7 using confocal microscopy after co-incubation for 24 h. Scale bar: 10 μm. (**H**-**J**) Fluorescence images of the healthy and UC mice (**H**), main organs (**I**), and gut tissues (**J**) at different time after oral administration of DiD-labeled SFELNVs

Efficient drug delivery strategy could enhance cellular uptake and concentration of drugs at the inflammatory site, which could improve the treatment of UC [19]. In order to estimate the cytotaxis and cellular uptake of SFELNVs by macrophages, we firstly established LPS-induced M1 macrophage pro-inflammatory phenotypes. We found that the expression of CD80 was upregulated with the increasing of the LPS concentration. When the LPS concentration reached 500ng/mL, the CD80 expression had no difference along with the LPS concentration increase by flow cytometry (Fig. 2A and B). While for CD86 expression, the LPS concentration need to reach 750ng/mL (Fig. 2C and D). Further, we investigated the mRNA expression of M1 phenotype marker iNOS using qPCR. Consist with results of CD80 expression, the optimal LPS concentration induced iNOS expression was 500ng/mL (Fig. 2E). Thus, we chose 500ng/mL LPS concentration for further study. Efficient drug uptake could decrease the adverse effect and dosage of drugs, we therefore incubated RAW264.7 with SFELNVs to assess the cell uptake of SFELNVs by macrophages in vitro. Flow cytometry analysis showed that the SFELNVs-labeled with the DiO was taken up by RAW264.7 cells, the quantified fluorescence intensity of DiO was 3.62%, 14.91%, 23.12%, 28.26%, and 45.83% at 12 h, 24 h, 36 h, 48 h and 72 h, respectively (Fig. 2F). Furthermore, the uptake capacity of SFELNVs by macrophages was also presented by confocal images. Compared to M0 phenotype RAW264.7, more DiO-labeled SFELNVs could be taken up by M1 phenotype macrophages, indicating SFELNVs could target M1 pro-inflammatory phenotype macrophages (Fig. 2G).

Oral administration has the advantage of convenient, economical and comfortable delivery strategy, and fewer side effects for patients, which is extensively used in the therapy of diseases [42, 43]. We next evaluated the gut-targeting ability of SFELNVs through oral administration. Colitis mice and healthy mice were intragastric administered with DiD-labeled SFELNVs. Fluorescent images for the whole-body, main organs, and digestive tract were obtained at 6 h, 12 h, 24 h, and 48 h after oral administration. Results showed that the highest fluorescence signals could be observed in NC + SFELNVs, UC + DiD, and UC + SFELNVs groups at 6 h in vivo. While there were strong DiD signals in UC + SFELNVs group at 12 h and 24 h compared to NC + SFELNVs and UC + DiD groups (Fig. 2H). The gut and vital organs (heart, liver, spleen, lung, and kidney) of mice were observed the distribution of SFELNVs after sacrificing the mice. Fluorescence images demonstrated that the SFELNVs were mainly distributed in the digestive tract, including gut, liver, and kidney, and the signals attenuated 48 h later, indicating that the SFELNVs were metabolized out of the body (Fig. 2I). Additionally, there were strong fluorescence signals of gut tissues at 6 h, indicating that the SFELNVs could be effectively taken up in gut. Compared to NC + SFELNVs and UC + DiD groups, the fluorescence signals were much stronger and longer retention time in UC + SFELNVs group (Fig. 2J). Collectively, the SFELNVs could be taken up by macrophages in vitro and had a favorable inflammation-targeting ability in vivo.

### SFELNVs could inhibit macrophage proliferation and inflammatory effect and promote M2 phenotype polarization in vitro

**Fig. 3:**
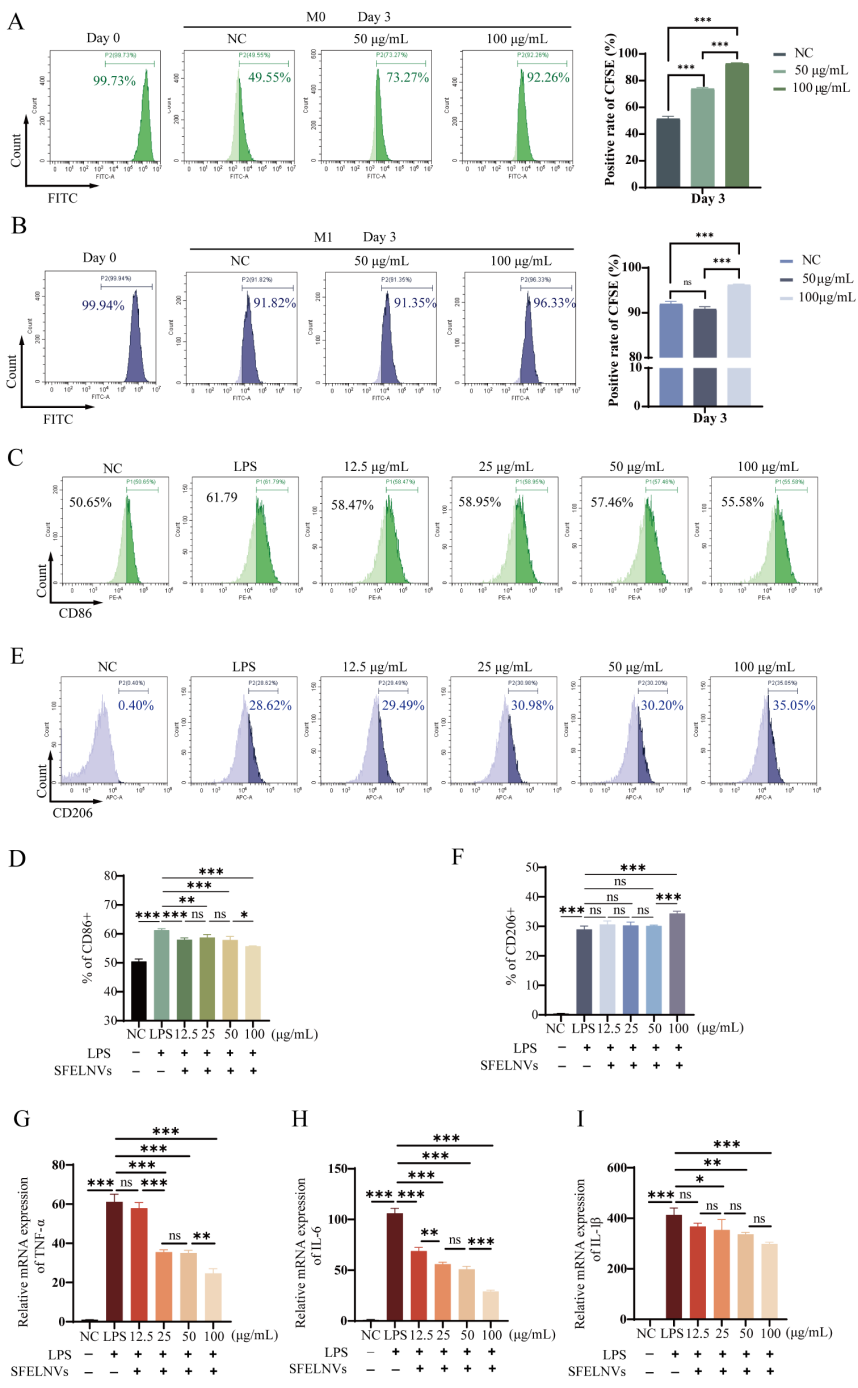
Investigation of the effects of SFELNVs on macrophage proliferation and polarization and anti-inflammatory function in vitro. (**A**-**B**) The proliferation CFSE-labeled RAW264.7 (M0 and M1 macrophage) was detected through flow cytometry when co-incubation with 50–100 µg/mL SFELNVs at day3. (**C**-**D**) CD86 expression were detected by flow cytometry after LPS-induced RAW264.7 cells co-incubation with different concentrations of SFELNVs. (**E**-**F**) CD206 expression were detected by flow cytometry after LPS-induced RAW264.7 cells co-incubation with different concentrations of SFELNVs. (**G**-**I**) Relative mRNA expression of pro-inflammatory factors, TNF-α (**G**), IL-6 (**H**), and IL-1β (**I**). *n* = 3, *, *p* < 0.05; **, *p* < 0.01; ***, *p* < 0.001, and ns, not significant

There were lots of macrophages in the whole gut tissues. They would enrich at inflammatory site with UC developing. Inhibiting the proliferation of macrophages would contribute to UC treatment. Thus, we explored the effects of SFELNVs on the macrophage proliferation. The fluorescence intensity analysis for the CFSE-labeled macrophage was acquired at day 3 after co-incubation with the SFELNVs via flow cytometry. We found that the quantified fluorescence intensity of CFSE-labeled M0 macrophage was 49.55%, 73.27%, and 92.26% at day3, and 91.82%, 91.35%, and 96.33% of CFSE-labeled M1 macrophage at day 3 after co-incubation with 0, 50, and 100 µg/mL SFELNVs, respectively (Fig. 3A and B). Thus, these results indicated that SFELNVs could inhibit the proliferation of macrophages.

Macrophage polarization is important for the development of gut inflammation. M1 phenotype produces pro-inflammatory factors and aggravates colitis. While M2 subtype plays the vital roles in gut homeostasis and barrier repair. Hence, promoting the transition of M1 to M2 phenotype is a critical strategy to treat UC. To evaluate the effects of SFELNVs on the macrophage polarization, we investigated the CD86 and CD206 expressions of LPS-induced RAW264.7 cells by flow cytometry. We found that 100 µg/mL SFELNVs could significantly inhibit CD86 expression (55.58%) (Fig. 3C and D) and enhance CD206 expression (35.05%) (Fig. 3E and F) compared to other groups. These results have shown that SFELNVs could boost the transition of M1 to M2 phenotype.

*Sophora Flavescens* have several pharmacological effects, including anti-inflammatory, oxidation resistance, and immunoregulation. SFELNVs contained some components of *Sophora Flavescens*, we therefore speculated that SFELNVs possessed similar anti-inflammatory functions. LPS-induced M1 phenotype macrophages were used to explore the function of SFELNVs in vitro. TNF-α, IL-1β, and IL-6, which produced by M1 phenotype macrophages, were upregulated in LPS-induced group compared to NC group by qPCR detection (Fig. 3G and I). Meanwhile, 100 µg/mL SFELNVs could significantly decrease pro-inflammatory factors TNF-α and IL-6 mRNA expressions (Fig. 3G and H). Notably, 25 µg/mL SFELNVs could inhibit IL-1β mRNA expression, and the concentration variation of SFELNVs had no different effect on IL-1β expression (Fig. 3I). Taken together, SFELNVs could inhibit the proliferation of macrophages and promote M2 phenotype polarization, and had effective anti-inflammatory function.

In order to explore the function of SFELNVs in inhibiting inflammation, we performed miRNA sequencing analysis and found 140 miRNAs enrichment in SFELNVs (Extended data Fig. 4A and B). And miRNA target genes were enriched in MAPK, WNT, Hippo pathways, and was related to the positive regulation of cell components biogenesis and focal adhesion (Extended data Fig. 4C and F), which was consistent with the results of network pharmacology. To investigate whether the function of SFELNVs in regulating macrophage polarization through miRNA, miR4371c was overexpressed in RAW264.7. We found that the expression of M1 marker (CD86) was 94.2%, 91.1%, and 82.8% in LPS group, miRNA-NC group, and miR4371c mimic group, respectively. In addition, the expression of M2 marker (CD206) was 0.62%, 0.15%, and 1.80% in LPS group, miRNA-NC group, and miR4371c mimic group using flow cytometry, respectively (Extended data Fig. 4G). These results indicated that miR4371c could decrease M1 marker (CD86) and increase M2 marker (CD206) expression. Thus, SFELNVs-contained miR4371c could promote M2 polarization.

### SFELNVs@CX5461 could promote macrophage apoptosis and anti-inflammatory effect in vitro

**Fig. 4:**
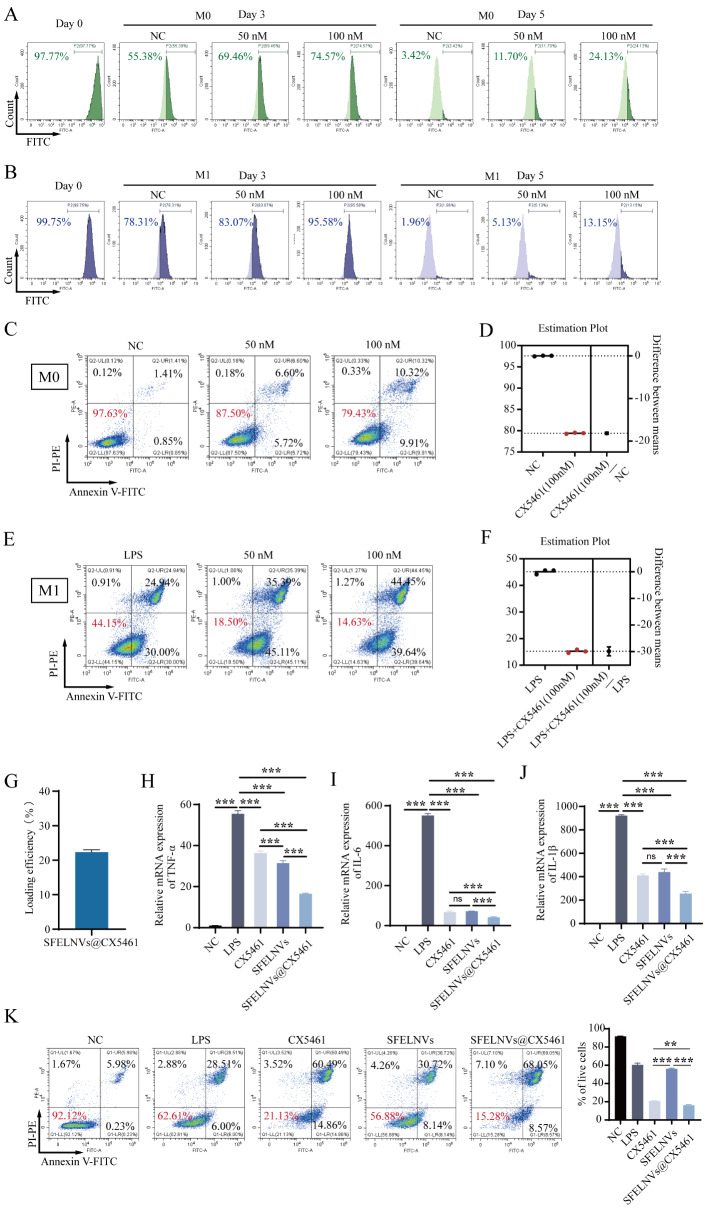
Evaluation of the effects of SFELNVs@CX5461 on macrophage apoptosis and anti-inflammatory function in vitro. (**A**) Proliferation of CFSE-labeled M0 RAW264.7 was detected by flow cytometry after co-incubation with 50 nM or 100 nM CX5461. (**B**) Proliferation of CFSE-labeled M1 RAW264.7 was detected by flow cytometry after co-incubation with 50 nM or 100 nM CX5461. (**C**-**D**) Apoptosis of M0 RAW264.7 was detected by flow cytometry after treatment with 50 or 100 nM CX5461 for 24 h. (**E**-**F**) Apoptosis of M1-type RAW264.7 was detected by flow cytometry after treatment with 50 or 100 nM CX5461 for 24 h. (**G**) Loading efficiency of the SFELNVs@CX5461 was detected by UV-spectrophotometer. (**H**-**J**) Relative mRNA expression of pro-inflammatory factors, TNF-α (**H**), IL-6 (**I**), and IL-1β (**J**). (**K**) Apoptosis of M1-type RAW264.7 was detected by flow cytometry after 24 h treatment with 100nM CX5461, 100 µg/mL SFELNVs, and SFELNVs@CX5461. *n* = 3, **, *p* < 0.01; ***, *p* < 0.001, and ns, not significant

In order to evaluate the role of CX5461 in UC, we firstly investigated the proliferation of M0 and M1 macrophage after CX5461 treatment. The fluorescence intensity analysis for the CFSE-labeled macrophage was acquired at day 3 and 5 after co-incubation with CX5461 through flow cytometry. Results have shown that the quantified fluorescence intensity of CFSE-labeled M0 macrophage was 55.38%, 69.46%, and 74.57% at day3, and 3.42%, 11.70%, and 24.13% at day 5 after co-incubation with 0, 50, and 100nM CX5461, respectively (Fig. 4A). Consistent with the results of M0 macrophage proliferation, we found that CX5461 could obviously inhibit M1 macrophage proliferation. The quantified fluorescence intensity was 78.31%, 83.07%, and 95.58% at day3, and 1.96%, 5.13%, and 13.15% at day 5 after co-incubation with 0, 50, and 100nM CX5461, respectively (Fig. 4B). Further, we explored the effects of CX5461 on the macrophage apoptosis. Compared with control group, the survival rate was 87.50% and 79.43% at 50 and 100nM CX5461 in M0 macrophages, respectively (Fig. 4C). The survival rate of M0 macrophages decreased by 18% (Fig. 4D). In LPS-induced M1 macrophages, the survival rate was 18.50% and 14.63% at 50 and 100nM CX5461, respectively (Fig. 4E). The survival rate of M1 macrophages decreased by 30% (Fig. 4F), indicating that CX5461 could effectively facilitate macrophages apoptosis.

To investigate the effects of the combination of SFELNVs and CX5461 on the anti-inflammatory effect in vitro, CX5461 was encapsulated into SFELNVs to confirm the secretion of pro-inflammatory cytokines. We firstly investigated the nanoparticle size and zeta potential, and the drug release rate of SFELNVs@CX5461 (Extended data Fig. 5). The nanoparticle size of SFELNVs@CX5461 was about 110 nm (Extended data Fig. 5A). The zeta potential of SFELNVs@CX5461 was around − 20mV (Extended data Fig. 5B). We then assessed the encapsulation efficiency of SFELNVs@CX5461 by UV-spectrophotometer. The encapsulation efficiency of SFELNVs@CX5461 was 23% (Fig. 4G). We also examined the release of encapsulated CX5461 from SFELNVs@CX5461 at different times (2 h, 6 h, 8 h, 10 h, 12 h, 18 h, 24 h, 36 h, 48 h) by Ultraviolet-visible spectroscopy (UV-Vis). The results suggested that with the increase of incubation time, CX5461 could be gradually released from SFELNVs with a peak time at 48 h. The drug release rate was 80% and 84% at 24 h and 48 h, respectively (Extended data Fig. 5C). We further evaluate the mRNA expression of pro-inflammatory factors, such as TNF-α, IL-1β, and IL-6. SFELNVs and CX5461 both could reduce the TNF-α, IL-6, and IL-1β expression by qPCR (Fig. 4H-J). Moreover, SFELNVs@CX5461 obviously inhibited TNF-α, IL-6, and IL-1β expression compared with other groups (Fig. 4H-J). In addition, we analyzed the effects of SFELNVs@CX5461 on the macrophage apoptosis by flow cytometry. We found that SFELNVs@CX5461 significantly boosted macrophage apoptosis (Fig. 4K). Therefore, there results have demonstrated that CX5461 was encapsulated into SFELNVs to synergistically facilitate macrophage apoptosis and enhance anti-inflammatory effect in vitro.

### Oral administration of SFELNVs@CX5461 could alleviate DSS-induced colitis in vivo

**Fig. 5:**
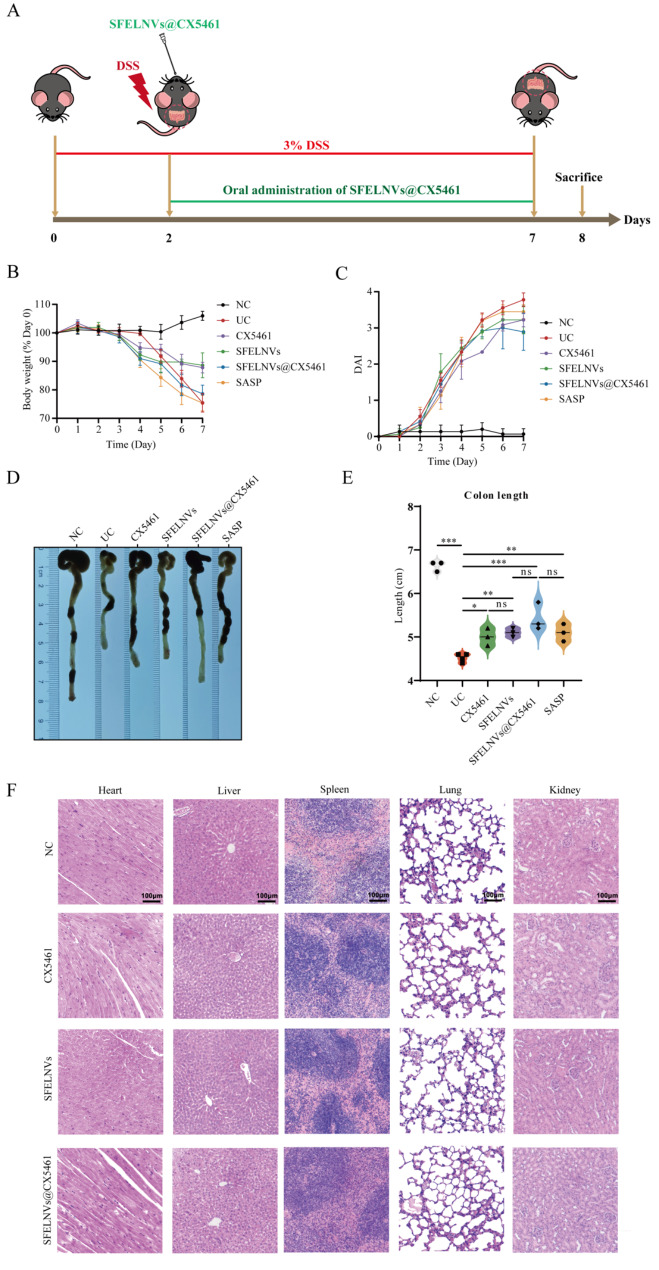
Oral administration of SFELNVs@CX5461 could alleviate DSS-induced colitis in vivo. (**A**) Illustration of the experimental design. (**B**) Daily body weight loss. (**C**) DAI scores. (**D**) Images of the colon length. (**E**) Quantitative analysis of changes in colon length. *n* = 3, *, *p* < 0.05; **, *p* < 0.01; ***, *p* < 0.001, and ns, not significant. (**F**) H&E staining of main organs (heart, liver, spleen, lung, and kidney) in each group. Scale bar: 100 μm

As mentioned above, SFELNVs were localized to the inflamed colon. The anti-inflammatory properties of SFELNVs@CX5461 made it promising for clinical application. We then used DSS-induced mice colitis to verify its anti-inflammatory activity further, and the experimental design illustration is shown in Fig. 5A. It was found that the body weight of DSS group mice decreased from the fourth day and decreased sharply from the fifth day. In contrast, oral administration of the SFELNVs@CX5461 and SFELNVs groups and injection of CX5461 group prevented the body weight loss to some extent compared to UC group (Fig. 5B). In addition to changes in body weight, the disease activity index (DAI) including stool consistency and fecal occult blood was also analyzed. As shown in Fig. 5C, compared with the UC group, the DAI of mice in the CX5461, SFELNVs, and SFELNVs@CX5461 groups increased slowly. The colon length also showed that the colon in DSS group mice was significantly shortened and recovered to a certain extent after CX5461, SFELNVs, and SFELNVs@CX5461 treatment compared with UC group (Fig. 5D and E).

To assess the biocompatibility of SFELNVs@CX5461 in vivo, the mice were oral administered daily with SFELNVs and SFELNVs@CX5461 at a dose of 80 mg/kg, and orally administered daily with CX5461 for 7 days. At the end of the experiment, after sacrificing the mice, the major organs (heart, liver, spleen, lung, and kidney) were collected for H&E staining. Histological sections also showed no pathological changes in organs in both SFELNVs@CX5461-treated mice and control mice (Fig. 5F), indicating the excellent biocompatibility of SFELNVs@CX5461. Collectively, oral administration of SFELNVs@CX5461 could alleviate acute colitis and possess good biosafety in vivo.

### Oral administration of SFELNVs@CX5461 could relieve inflammation in vivo

**Fig. 6:**
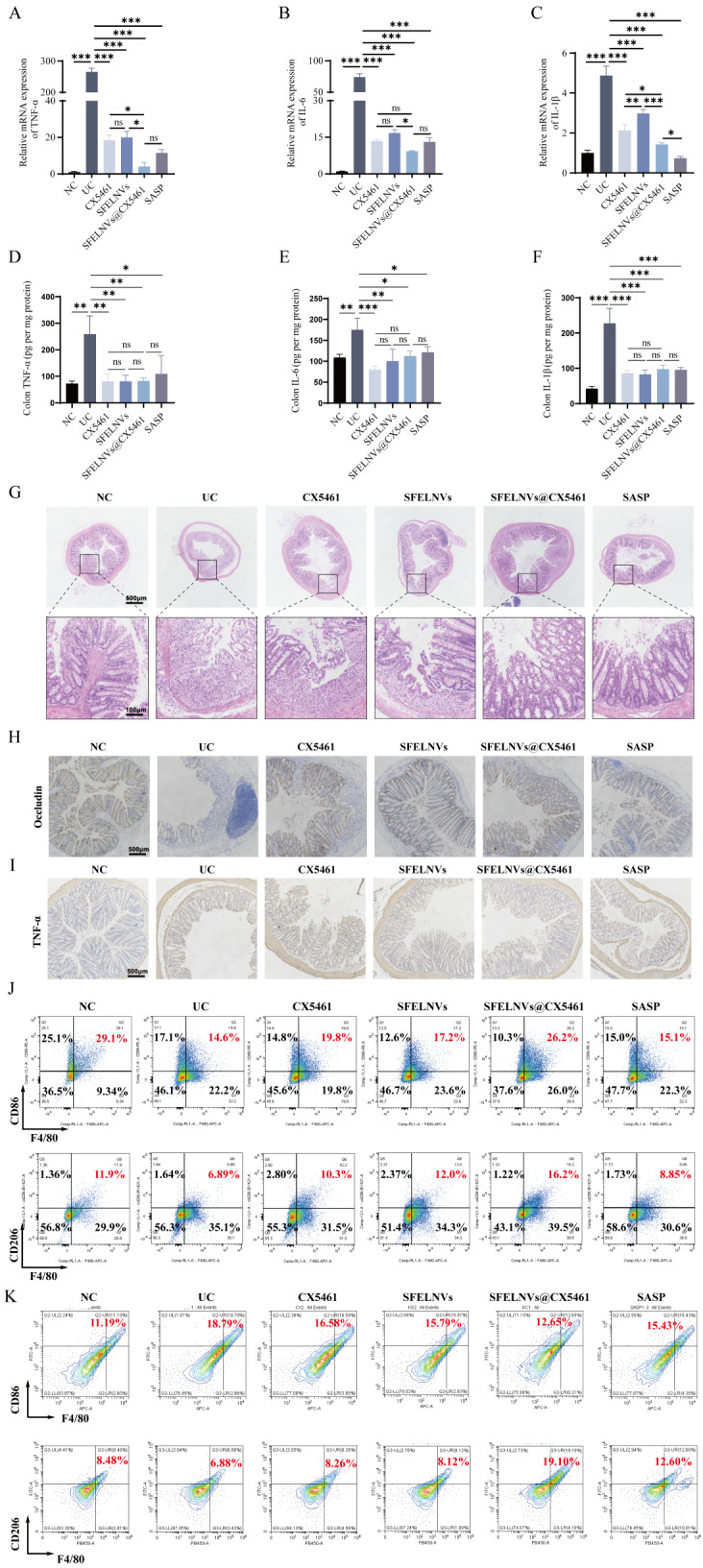
Oral administration of SFELNVs@CX5461 could relieve colonic inflammation in vivo. (**A**-**C**) Relative mRNA expression of pro-inflammatory factors TNF-α (A), IL-6 (**B**), and IL-1β (**C**) in colon tissue by qPCR. (**D**-**F**) Relative protein expression of pro-inflammatory factors TNF-α (**D**), IL-6 (E), and IL-1β (**F**) in colon tissue by ELISA. *n* = 3, *, *p* < 0.05; **, *p* < 0.01; ***, *p* < 0.001, and ns, not significant. (**G**) H&E staining of colonic sections. Scale bar: 500/100µm. (**H**-**I**) IHC staining of colonic sections to detect the expression of Occludin (**H**) and TNF-α (**I**), Scale bar: 500 μm. M1 and M2 macrophages in spleen (**J**) and intestines (**K**) were detected by flow cytometry

During UC development, the secretion of pro-inflammatory cytokines induced the intestinal inflammatory response and leading to increased damage. Therefore, pro-inflammatory cytokines played an important role in colitis. The results of inflammatory factors showed that the mRNA expression levels of pro-inflammatory factors (TNF-α, IL-6, and IL-1β) in the DSS-treated group were significantly upregulated compared with the control group though qPCR (Fig. 6A and C). When treated with SFELNVs@CX5461, SFELNVs, and CX5461, the mRNA expression level of TNF-α, IL-6, and IL-1β was significantly decreased compared with the DSS group. Compared with SFELNVs and CX5461 group, SFELNVs@CX5461 could dramatically downregulate the mRNA expression of TNF-α, IL-6, and IL-1β (Fig. 6A and C). Remarkably, there was no significant difference in the mRNA expression of IL-6 between CX5461 and SFELNVs@CX5461 (Fig. 6B). Furthermore, we evaluated the protein expression of TNF-α, IL-6, and IL-1β by ELISA. We found that the protein expression of TNF-α, IL-6, and IL-1β in the DSS-treated group were significantly upregulated compared with the control group (Fig. 6D and F). The expression level of TNF-α, IL-6, and IL-1β was significantly decreased in SFELNVs, CX5461, and SFELNVs@CX5461 group, while the expression of pro-inflammatory factors was no significant difference in SFELNVs, CX5461, and SFELNVs@CX5461 group (Fig. 6D and F). Additionally, histological staining with H&E was used to observe the injury and lesion of colon tissue. Results showed that the colonic mucosa of healthy mice in the control group was intact, and the crypt was apparent (Fig. 6G). In contrast, crypts disappeared, inflammatory cells infiltrated, and epithelial erosion in colonic tissues of mice with colitis (Fig. 6G). Compared with the DSS group, the colonic tissue of the SFELNVs@CX5461 treatment group recovered, and the morphology was similar to that of the healthy control group (Fig. 6G). Occludin played essential roles in maintaining cell-cell junction and gut barrier. Thus, IHC was used to observe Occludin and TNF-α of colon tissues. We found that Occludin expression was deregulated in DSS-induced colitis compared with the healthy mice. Oral administration of SFELNVs@CX5461 could upregulated the expression of Occludin (Fig. 6H). In contrast, TNF-α expression was upregulated in DSS-induced colitis compared with the healthy mice. Oral administration of SFELNVs@CX5461 could decrease the expression of TNF-α (Fig. 6I).

Additionally, we also estimated the effects of SFELNVs@CX5461 on macrophages of spleen and colitis site by flow cytometry (Fig. 6J and K). Flow cytometry have shown that both the proportion of spleen M1 and M2 macrophages were significantly decreased in DSS-induced UC group, speculating that M1 and M2 macrophages might be recruited to the intestinal inflammation site. And the proportion of spleen M2 macrophages was increased in SFELNVs@CX5461 treatment group (Fig. 6J). Expectedly, with consistent result in vitro, DSS could increase M1 macrophages proportion in colitis mice. And the M1 macrophages proportion was reduced, and M2 macrophages proportion was upregulated after SFELNVs@CX5461 treatment (Fig. 6K). Our results demonstrated that orally administrated SFELNVs@CX5461 possessed anti-inflammatory activity, and could relieve DSS-induced inflammation in mice.

## Discussion

Oral administration drug mesalazine and SASP are the first-line medicine for UC therapy. However, some patients would generate 5-ASA intolerance [44]. Close to 15% of patients need surgical therapy after 5-ASA treatment fails [45]. Small molecular-targeted therapy is an excellent approach for UC patients. However, expensive cost limits its application [46]. Other conventional drugs, they were administrated by systemic administration or intravenous injection, resulting in side effects and tissue toxicity [32]. Thus, convenient and safety strategy for UC needs to develop. Recently, compared to artificial nanoparticles, natural cell membrane or bacteria-derived vesicles and plant-derived exosomes-like nanovesicles paid more and more attentions. Results have shown that bacteria-derived vesicles could serve as vaccine to perform anticancer response [47, 48]. However, avoiding degradation of bacteria-derived vesicles is currently a major predicament by oral administration to treat UC. Plant-derived exosomes-like nanovesicles possess the function of oral administration, excellent stability, and targeting inflammatory sites, and therefore possess the potential of UC treatment [19, 32]. In this study, we screened the excellent delivery platform in the previous experiments. We chose five traditional Chinese medicines with anti-inflammatory functions, including *Sophora Flavescens*, *Codonopsis Pilosula*, *Astragalus Membranaceus*, *Andrographis Paniculate*, and *Bupleurum Chinense*. We found that *Sophora Flavescens*-derived exosomes-like nanovesicles (SFELNVs) were more stable than other four nanovesicles (Extended data Fig. 1), and SFELNVs were efficiently taken up by colonic epithelial cells and inflammatory subtype macrophage (Extended data Fig. 2). Indeed, oral administration of SFELNVs possess targeting ability to the inflamed colon, excellent stability, and anti-inflammatory effects (Figs. 2 and 3). These results were similar with other plant-derived exosomes-like nanovesicles, such as turmeric [32]. Dissimilarly, SFELNVs also inhibited macrophage proliferation and promoted M2 phenotype polarization (Fig. 3), thereby mitigating inflammatory progression. Furthermore, we also analyzed the function of SFELNVs in anti-inflammation using LC-MS and miRNA sequencing (Extended data Fig. 3 and Extended data Fig. 4). We found that SFELNVs contained some anti-inflammatory compounds. Moreover, flow cytometry showed that miR4371c could promote M2 polarization. Thus, we hold the opinion that compounds and miR4371c in SFELNVs synergistically involved in the anti-inflammatory function of SFELNVs.

CX5461, an orally administrated rRNA synthesis inhibitor, exhibited effective anticancer and anti-inflammation activity [23, 49, 50]. Recently, CX5461 possessed immunosuppressive functions in allograft models [24] and atopic dermatitis [25]. In this study, we found that CX5461 could decrease macrophages proliferation, enhance macrophages apoptosis, and repress inflammatory cytokine expression (Fig. 4), indicating that CX5461 possess anti-inflammatory function. However, low solubility, acute toxicities in low pH and clinical intravenous injection limited its application in colitis. We thus loading CX5461 into SFELNVs by electroporation. SFELNVs@CX5461 with 80 mg/kg treatment showed significantly anti-inflammatory activity and the most extended colon length in UC mice. We also found that SFELNVs@CX5461 could obviously inhibit pro-inflammatory factors expression in LPS-induced M1 macrophages and UC mice, indicating that SFELNVs@CX5461 could serve as a potential UC therapeutic drug. In addition, we assessed the macrophages subtypes in spleen and colitis site after SFELNVs@CX5461 treatment (Fig. 6J and K). We found that both the proportion of spleen M1 and M2 macrophages were significantly decreased in DSS-induced UC group (Fig. 6J), this phenomenon is not consistent with results *in vitro.* We surmised duo to the spleen far away from the inflammatory site of the intestine, and thus the polarization of macrophages in the inflammatory site may not be consistent with the polarization of macrophages in the spleen. On the other hand, M1 and M2 macrophages might be recruited to the intestinal inflammation site, thereby the proportion of M1 and M2 macrophages in spleen was reduced. Additionally, in previous studies, CX5461 could facilitate Treg differentiation and infiltration [51], and inhibit T cells infiltration [23] and activation [52]. Treg and T cells are involved in intestinal homeostasis [53, 54]. Thus, whether SFELNVs@CX5461 regulates Treg and T cell infiltration needs further studies in mice colitis.

In summary, we designed a novel and nontoxic *Sophora Flavescens*-derived exosome-like nanovesicle that could localize to the inflamed colon and possesses anti-inflammation functions through oral administration (Scheme 1). SFELNVs could be efficiently taken up by RAW 264.7 cells. MiR4371c in SFELNVs could facilitate M2 macrophage polarization, thereby reducing inflammation. In addition, SFELNVs@CX5461 could enhance anti-inflammatory effects in vitro and in vivo to alleviate UC. Thus, SFELNVs@CX5461 represents a targeted therapeutic strategy to UC with excellent biocompatibility and stability.

## Electronic supplementary material

Below is the link to the electronic supplementary material.

Supplementary Material 1

## Data Availability

No datasets were generated or analysed during the current study.

## Abbreviations

UC: Ulcerative Colitis
TNF-α: Tumor Necrosis Factor-α
IL-1β: Interleukin-1β
IL-6: Interleukin-6
IBD: Inflammatory Bowel Disease
CRC: Colorectal Cancer
SASP: Sulfasalazine
iNOS: Inducible Nitric Oxide Synthase
NFAT: Nuclear Factor of Activated T Cells
DNCB: 2,4-Dinitrochlorobenzene
PELNPs: Plant-Derived Exosomes-Like Nanoparticles
FBS: Fetal Bovine Serum
LPS: Lipopolysaccharides
DiO: 3,3′-Dioctadecyloxacarbocyanine Perchlorate
DiR: 1,1-Dioctadecyl-3,3,3,3-Tetramethylindotricarbocyaine Iodide
DiD: 1,1’-Dioctadecyl-3,3,3’,3’-Tetramethylindodicarbocyanine, 4 Chlorobenzenesulfonate Salt
IHC: Immunohistochemistry Staining
TEM: Transmission Electron Microscopy
DLS: Dynamic Light Scattering
NTA: Nanoparticle Tracking Analysis
CFSE: Carboxyfluorescein Succinimidyl Ester
HE: Hematoxylin & Eosin staining
DAB: 3,3 N-Diaminobenzidine Tertrahydrochloride
SGF: Simulation of Gastric Fluid
SIF: Simulation of Intestinal Fluid
SCF: Simulation of Colonic Fluid
PPI: Protein-Protein Interaction Networks
MAPK: Mitogen-Activated Protein Kinase
mTOR: mechanistic Target of Rapamycin Kinase
STAT1: Signal Transducer and Activator of Transcription 1
GO: Gene Ontology
KEGG: Kyoto Encyclopedia of Genes and Genomes
DAI: Disease Activity Index
5-ASA: 5-Aminosalicylic Acid
