# Supplementary material for: Oral administration of Sophora Flavescens-derived exosomes-like nanovesicles carrying CX5461 ameliorates DSS-induced colitis in mice
Source: J Nanobiotechnology. 2024 Oct 8;22:607. doi: 10.1186/s12951-024-02856-z (PMC11463058; doi:10.1186/s12951-024-02856-z)
Supplement: Supplementary file 1 — Supplementary Material 1 [file 12951_2024_2856_MOESM1_ESM.docx]

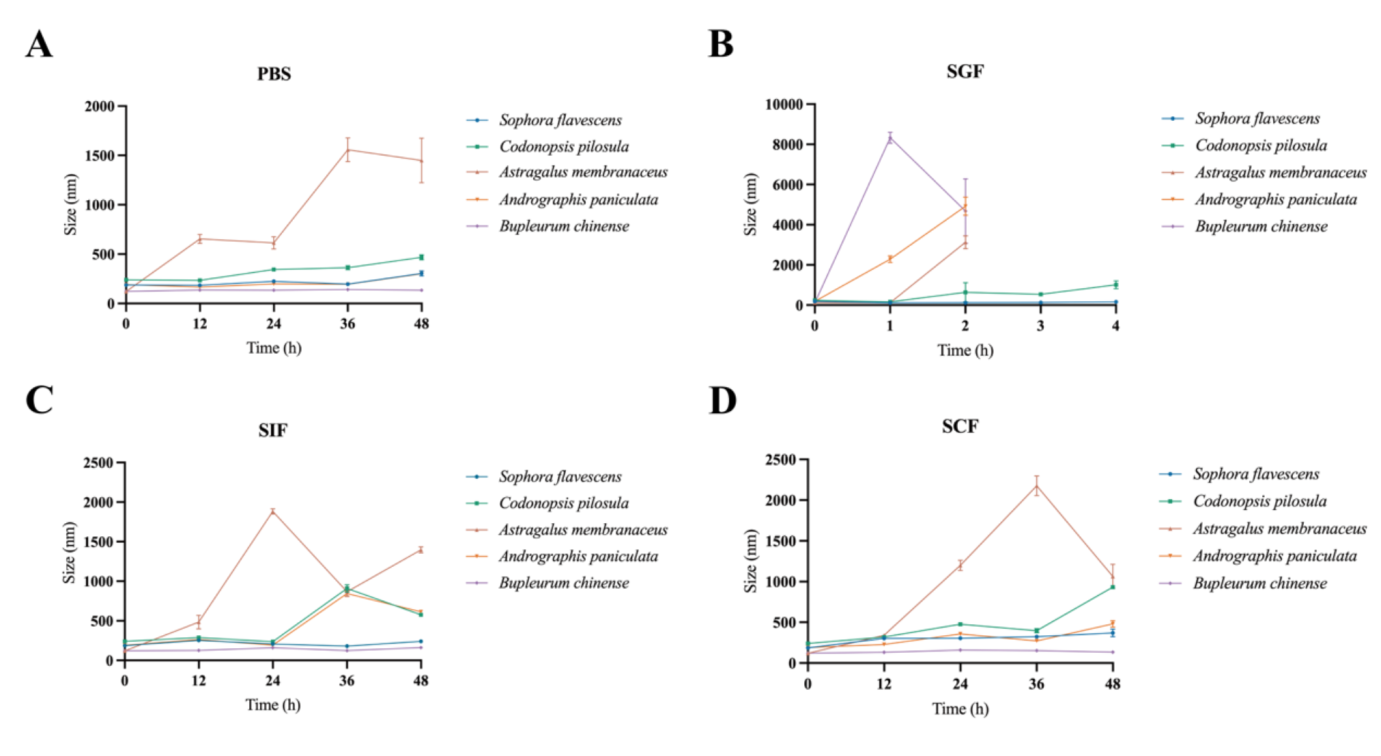
 **Extended data Fig. 1. The stability of plant-derived exosomes-like nanoparticles (PELNPs) in gastric and intestinal simulation.** (A-D) Five PELNPs (*Sophora Flavescens*, *Codonopsis Pilosula*, *Astragalus Membranaceus*, *Andrographis Paniculate*, and *Bupleurum Chinense*) were treated in PBS (A), simulation of gastric fluid (SGF) (B), simulation of intestinal fluid (SIF) (C), and simulation of colonic fluid (SCF) (D) *in vitro.* The particle diameters were detected by Malvern Zetasizer Pro.


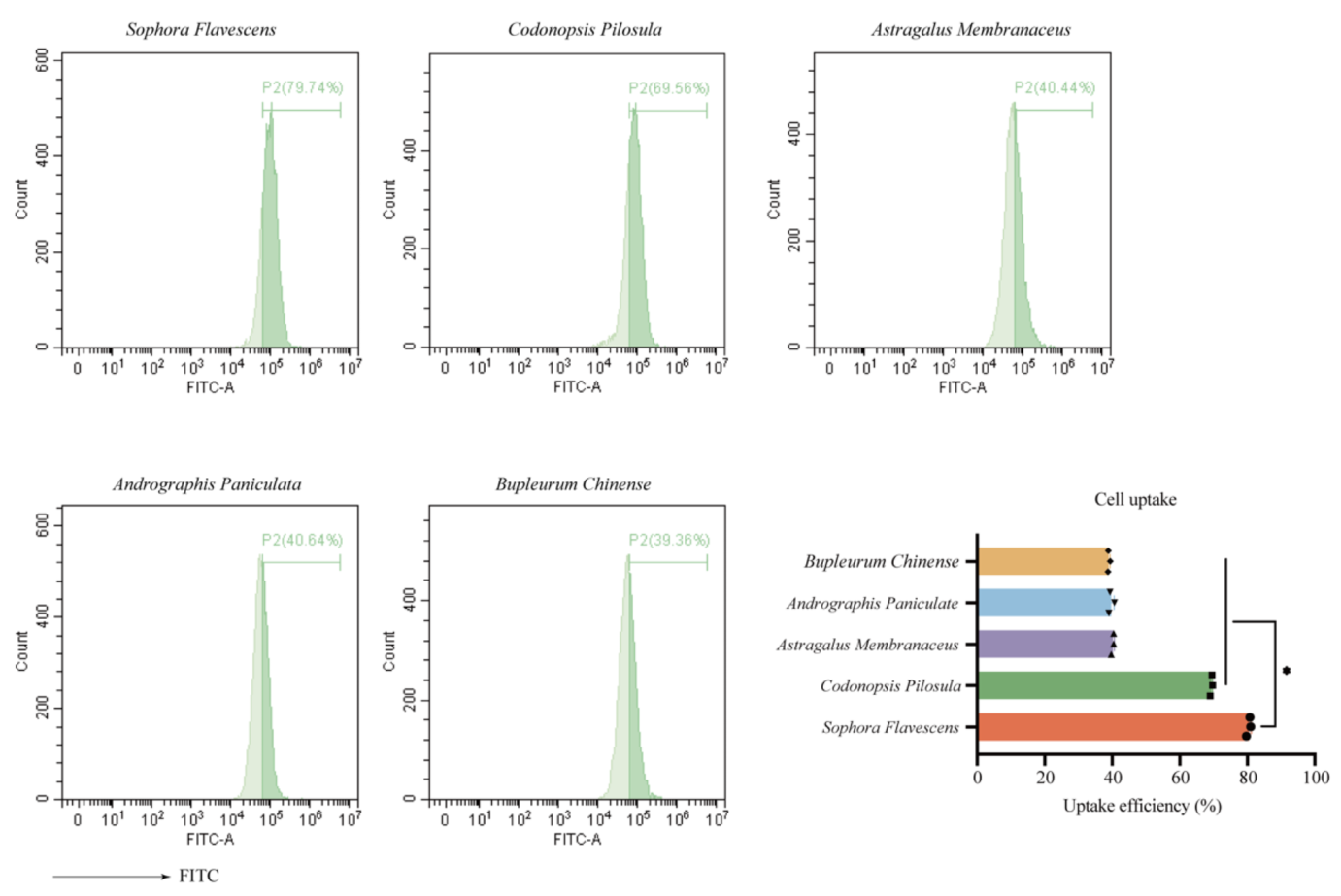


**Extended data Fig. 2. Uptake of the five PELNPs in Caco-2 cells.** The uptake efficiency of the five PELNPs-labeled with the DiO in Caco-2 cells via flow cytometry. Caco-2 cells were treated by 200μg/mL *Sophora Flavescens*, *Codonopsis Pilosula*, *Astragalus Membranaceus*, *Andrographis Paniculate*, and *Bupleurum Chinense*-derived exosomes-like nanoparticles for 12h.


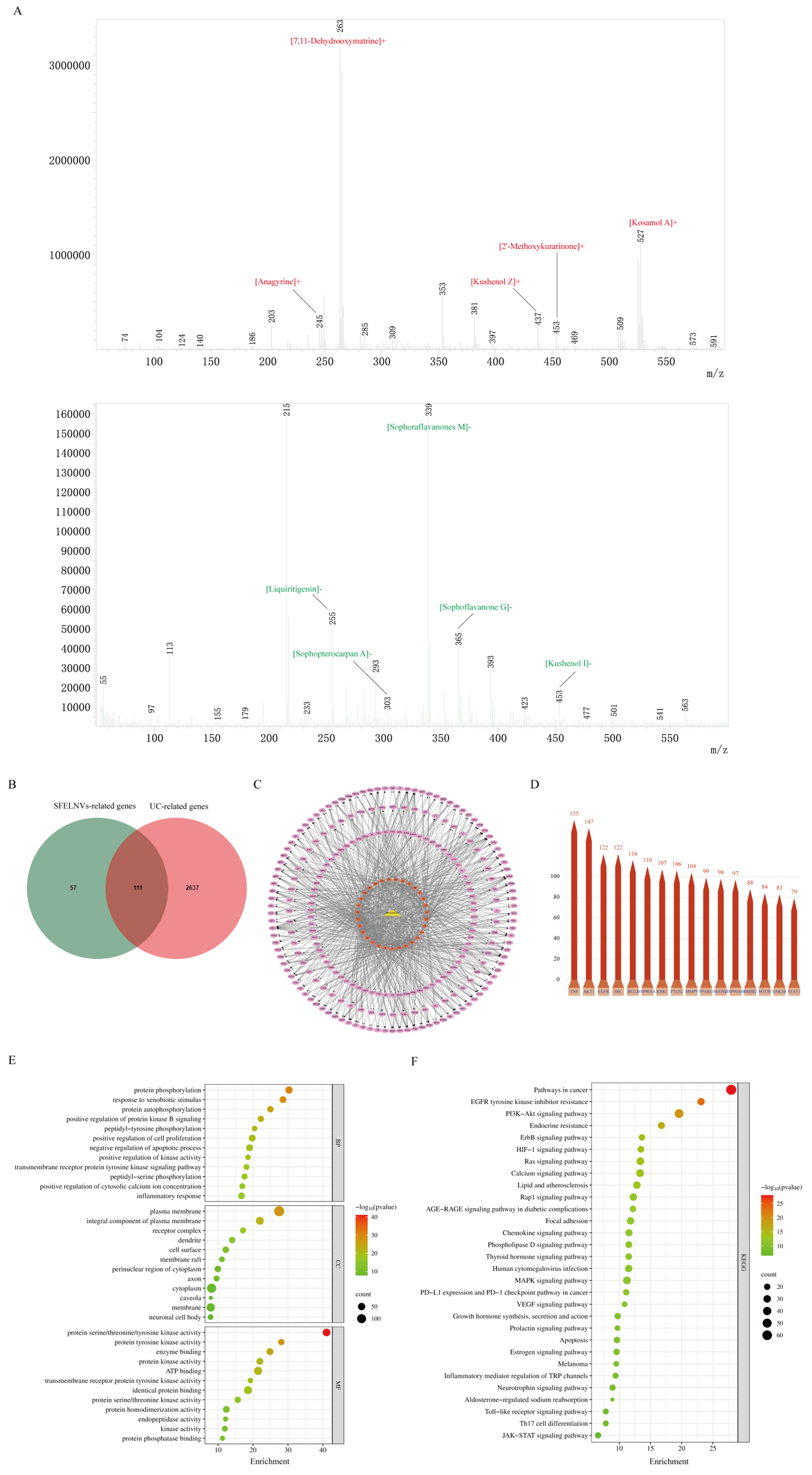


**Extended data Fig. 3. Network pharmacology analyzed the common target genes and pathways of SFELNVs and UC.** Investigation the component of SFELNVs through LC-MS (A). The Venn diagram of common target genes in SFELNVs and UC were identified using TCMSP, OMIM, TDD, and GeneCards online database (B). Analysis of protein interaction of the common target genes (C) and the top ten genes in the common target genes (D) using String database and Cytoscape. The functions of the common target genes were analyzed using GO (E) and KEGG enrichment (F) in DAVID database.


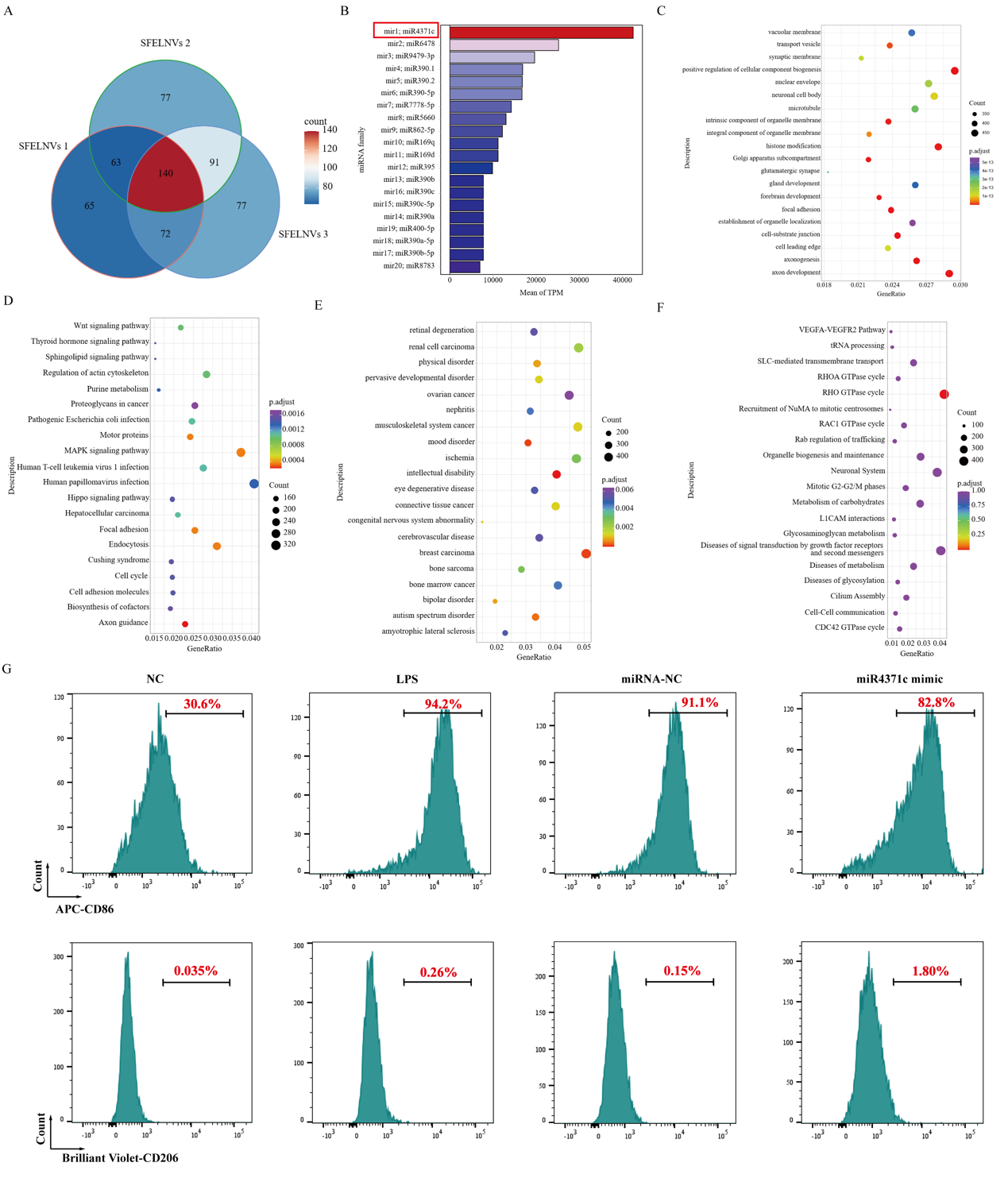


**Extended data Fig. 4. Analysis of the components of SFELNVs via miRNA sequencing and investigation miRNA4371c function by flow cytometry.** The Venn diagram of miRNAs in SFELNVs were identified using miRNA sequencing analysis (A). The list of the enrichment of top 20 miRNAs (B). The functions of the 140 miRNAs were analyzed using GO (C), KEGG (D), DO (E), and Reactome enrichment (F). Investigation the effects of miR4371c on the macrophage polarization (G).


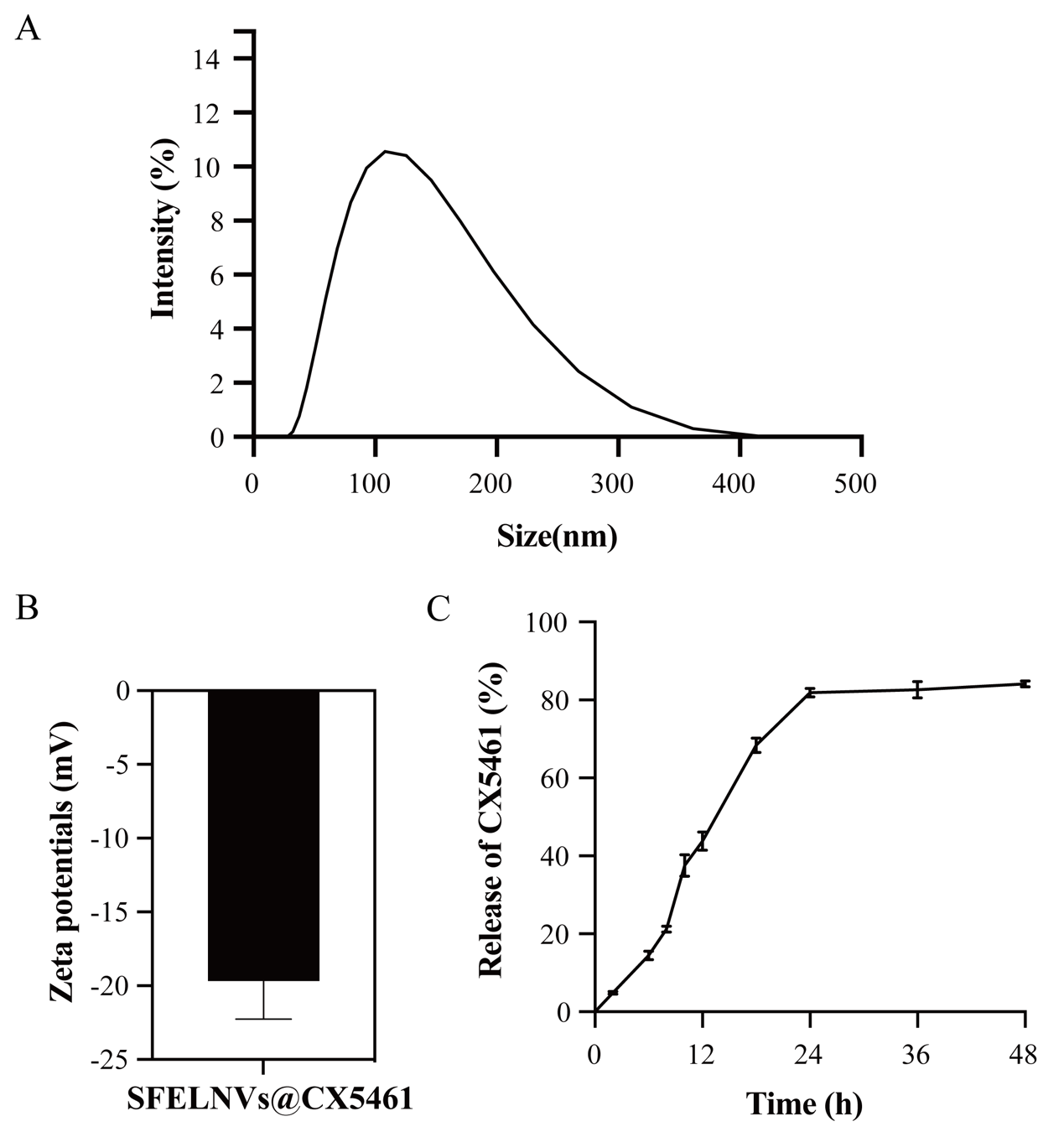


**Extended data Fig. 5. Characterization of SFELNVs@CX5461.** (A) The particle size distribution of SFELNVs@CX5461 was determined by the DLS. (B) Zeta potential of SFELNVs@CX5461 (n = 3). (C) The drug release rate of encapsulated CX5461 from SFELNVs@CX5461 at different times.
